# Strategies to prevent anthracycline-induced cardiotoxicity in cancer survivors

**DOI:** 10.1186/s40959-019-0054-5

**Published:** 2019-12-02

**Authors:** Neha Bansal, M. Jacob Adams, Sarju Ganatra, Steven D. Colan, Sanjeev Aggarwal, Rudolf Steiner, Shahnawaz Amdani, Emma R. Lipshultz, Steven E. Lipshultz

**Affiliations:** 1 Division of Pediatric Cardiology, Children’s Hospital at Montefiore, Bronx, NY USA; 2 Department of Public Health Sciences, University of Rochester School of Medicine and Dentistry, Rochester, NY USA; 3 Cardio-Oncology Program, Division of Cardiovascular Medicine, Department of Medicine, Lahey Hospital and Medical Center, Burlington, MA USA; 4 Cardio-Oncology Program, Dana-Farber Cancer Institute / Brigham and Women’s Hospital, Boston, MA USA; 5 Department of Pediatric Cardiology, Boston Children’s Hospital, Boston, MA USA; 6 Division of Pediatric Cardiology, Department of Pediatrics, Children’s Hospital of Michigan, Detroit, MI USA; 7 University of Zurich, Zurich, Switzerland; 8 Division of Pediatric Cardiology, Cleveland Clinic Children’s Hospital, Cleveland, OH USA; 9 Dana-Farber Cancer Institute, Boston, MA USA; 10 University of Miami Miller School of Medicine, Miami, FL USA; 11 Department of Pediatrics, University at Buffalo Jacobs School of Medicine and Biomedical Sciences, Oishei Children’s Hospital, 1001 Main Street, Buffalo, NY 14203 USA; 12 Oishei Children’s Hospital, Buffalo, NY USA; 13 Roswell Park Comprehensive Cancer Center, Buffalo, NY USA

**Keywords:** Cardiotoxicity, Cardio-oncology, Pediatrics, Beta-blockers, ACE inhibitors, Anthracyclines, Cancer

## Abstract

Cancer diagnostics and therapies have improved steadily over the last few decades, markedly increasing life expectancy for patients at all ages. However, conventional and newer anti-neoplastic therapies can cause short- and long-term cardiotoxicity. The clinical implications of this cardiotoxicity become more important with the increasing use of cardiotoxic drugs. The implications are especially serious among patients predisposed to adverse cardiac effects, such as youth, the elderly, those with cardiovascular comorbidities, and those receiving additional chemotherapies or thoracic radiation. However, the optimal strategy for preventing and managing chemotherapy-induced cardiotoxicity remains unknown. The routine use of neurohormonal antagonists for cardioprotection is not currently justified, given the marginal benefits and associated adverse events, particularly with long-term use. The only United States Food and Drug Administration and European Medicines Agency approved treatment for preventing anthracycline-related cardiomyopathy is dexrazoxane. We advocate administering dexrazoxane during cancer treatment to limit the cardiotoxic effects of anthracycline chemotherapy.

## Introduction

The number of cancer survivors continues to increase as the success of cancer treatment regimens improves. The number of people in the US living after cancer is diagnosed to be 16.9 million as of Jan 2019 and are projected to increase to 21.7 million by 2029 [1]. In the early 1970s, 12% of children who survived cancer of any type died within 15 years of diagnosis; by the early 1990s, the proportion had decreased to 6% [2, 3].

Anthracyclines are still the chemotherapeutic drug class of choice for treating many cancers [4]. That fact remains the case, even with the introduction of a multitude of new cancer therapies over the past several years, such as targeted drugs and immunotherapies, with improvement in the overall morbidity and mortality of several different cancers [5–7]. Although many anti-neoplastic therapies are cardiotoxic, anthracycline-induced cardiomyopathy and heart failure (HF) are prototypical and the most thoroughly studied. In fact, the evidence increasingly shows that cancer and cardiovascular disease (CVD) are linked through common risk factors in both pediatric patients and in an aging population as well as through the adverse cardiovascular effects of cancer treatment.

Cardiovascular disease in general, and HF in particular, appear strongly related to cancer [8]. They share classical risk factors, including smoking, sedentary lifestyle, and obesity. Immunologic responses are critically important in cardiac remodeling and may have strong implications for the physiology of tumors. Immune checkpoint inhibitors have greatly improved clinical outcomes in several cancers, but they have also been implicated in several dozen reported cases of fulminant myocarditis, many of which resulted in death [9].

Early medical therapy for HF has greatly improved outcomes in the general population because such therapy can reverse, ameliorate, or prevent progressive left ventricular (LV) dysfunction [10]. Commonly used neurohormonal blocking drugs, such as angiotensin converting enzyme inhibitors (ACEI), angiotensin receptor blockers (ARB), beta-blockers (BB), and aldosterone antagonists, reduce mortality or reverse LV remodeling in patients with non-cancer-related HF or with asymptomatic LV dysfunction. Their use has been explored in patients with chemotherapy-induced HF [11, 12]. In fact, chemotherapy-induced cardiac dysfunction and symptomatic HF, in both children and adults, is now usually treated according to general cardiology guidelines for HF. However, evidence in support of using these drugs to treat, let alone prevent, HF caused by chemotherapy-induced cardiotoxicity is lacking. Furthermore, there is no clear consensus on the right time to administer these drugs. Most of the current cardio-oncology recommendations to prevent cardiotoxicity are largely based on opinion [13]. Increasingly, these medications are being investigated for preventing cardiotoxicity by administering them during cancer therapy. Physicians caring for survivors of cancer, especially childhood cancers, need to be aware of the benefits and the potential pitfalls of these medications if they are to make informed decisions for their patients [14].

Despite the cardiotoxicity associated with anti-neoplastic therapy, improperly discontinuing, interrupting, or reducing this therapy to avoid cardiotoxicity may lead to poorer overall outcomes. We found no studies reporting that reducing chemotherapy in patients with asymptomatic LV dysfunction leads to a better quality-of-life over a lifespan by providing oncologic efficacy on one hand and minimizing toxicities and late effects on the other [15, 16]. However, identification of risk factors of cardiotoxicities in childhood cancer survivors via close surveillance is imperative to offer the preventive strategies currently available [17].

In this review, we summarize the primary and secondary strategies for preventing cardiotoxicity in cancer patients, especially that caused by anthracycline-containing cancer therapy.

## Characteristics of cardiotoxicity

One of the most common manifestations of cardiotoxicity is LV dysfunction. However, the exact definitions of cardiotoxicity and a “significant” reduction in cardiac function are controversial [18]. The first controversy concerns the threshold of clinically important LV dysfunction. Current guidelines based on the criteria of the American Society of Echocardiography define cardiotoxicity as a ≥ 10% drop in left ventricular ejection fraction (LVEF) from baseline or an absolute value of ≤53% [19]. According to the European Society of Medical Oncology’s Clinical Practice Guidelines Cardiac Review and Evaluation Committee [20], LV dysfunction is defined by “a) decrease in cardiac LVEF that is either global or more severe in the septum; b) symptoms of HF; c) signs of HF, including but not limited to the presence of a S3 gallop, tachycardia, or both; and d) a decline in LVEF of ≥5% to ≤55% with accompanying signs or symptoms of HF or a decline in LVEF of ≥10% to ≤55% without accompanying signs or symptoms.” According to a 2016 European Society of Cardiology Position Paper, echocardiography is the method of choice for detecting myocardial dysfunction before, during, and after cancer therapy. The paper defines cardiotoxicity as a decrease in LVEF of > 10% to a value ≤50%, which is defined as the lower limit of normal [21].

The second controversy is how LV dysfunction is measured. The introduction of newer cardiac imaging technologies, such as cardiac magnetic resonance imaging (cMRI) and strain by echocardiography or cMRI, that can detect asymptomatic LV dysfunction, has led to the realization that the incidence of anthracycline-induced cardiotoxicity is substantially higher than previously thought when measured with echocardiography alone [22]. This unexpected incidence is even higher when assessed with serum cardiac biomarkers, which are validated surrogate endpoints for late echocardiographic evidence of cardiotoxicity in long-term survivors of childhood cancer treated with anthracyclines, but concentrations of these biomarkers may also be elevated despite clinically unimportant cardiac damage [23].

The third controversy is that none of these definitions or screening measures for LV dysfunction capture the damage anthracyclines and other anti-neoplastic drugs cause to the heart that manifest in ways other than LV systolic dysfunction, such as atherosclerotic disease, diastolic dysfunction, and intracardiac conduction abnormalities. Nevertheless, at present, the most frequently used modalities for detecting cardiotoxicity are the periodic measurement of LVEF by echocardiography or cMRI, with echocardiography being the predominant technology for screening cancer patients [20]. The American Society of Clinical Oncology guidelines recommend continuous surveillance of all adults with a history of cancer treatment by means of a thorough history and physical and serial echocardiograms or cMRIs (in the case of poor echocardiographic images) in patients considered at high-risk for cardiotoxicity [24]. Newer imaging technologies, such as echocardiographic myocardial strain and strain rate, can also detect toxicity before abnormalities in the LVEF become apparent [16]. The American Society of Echocardiography and the European Association of Cardiovascular Imaging Guidelines recommend assessing global longitudinal strain as a routine component of clinical echocardiographic exams in adults at risk for cardiotoxicity [25]. No such guidelines exist for children. However, these modalities depend on LV loading conditions and heart rate, which are often disturbed in cancer patients [16]. Indicators of LV function provided by imaging modalities, including LVEF, decline during therapy and are poor predictors of chronic cardiomyopathy after therapy [16]. Thus, relying on monitoring modalities to modify lifesaving chemotherapy doses may do more harm than good in cancer patients without clinical cardiovascular symptoms of HF. A large unknown is the effect of cardiac monitoring and consequent dose reductions on cancer cure rates [15]. In spite of published guidelines, whether therapeutic decisions based on asymptomatic changes in LV function will improve overall survival or quality-of-life for these patients is unknown. Therefore, we do not support altering oncologic therapy based on echocardiographic changes in cancer patients without symptomatic cardiac disease.

Practice guidelines may affect clinical practice. Although they are imperfect and are a work-in-progress, they remain the cornerstone for informing clinical decisions [26]. However, to provide the most accurate state of the existing data on which practice guideline are developed the authors must delineate the quality of available data that support these guidelines. Further efforts evaluating longitudinal data and testing practice guidelines prospectively remain critical given the possibility that they might be causing harm.

## Principles for preventing cardiotoxicity

The relationship between cancer and CVD is bidirectional [27]. Well known risk factors for CVD, such as tobacco use, obesity, physical inactivity, poor nutrition, diabetes, excessive alcohol consumption, hypertension, and hyperlipidemia, are also risk factors for cancer [8]. An individual’s cardiovascular risk factors, such as hypertension, in a cancer survivor appeared to carry greater risk than the same risk factors in an individual without a history of cancer [28]. Additionally, each additional risk factor in a cancer survivor appears to impart a more-than-additive increase in the risk of CVD [29, 30]. Further, cancer survivors are more likely to have traditional cardiovascular risk factors than are their age-matched healthy controls [31]. Cancer patients with pre-existing CVD or cardiovascular risk factors are at higher risk for cardiac complications [32]. As a result, the traditional CVD risk factors cited above must be aggressively managed in this high-risk population, regardless of the type of cancer or treatment, to minimize the risk of adverse outcomes [32]. Further, cholesterol metabolites activate estrogen receptors and stimulate breast tumor growth [33]. Thus, lowering cholesterol levels through lifestyle modifications, medication, or exercise—all proven cardioprotective interventions—may also reduce the risk of breast cancer or at least slow the rate of tumor growth [34, 35].

Recent epidemiological studies, like a large Danish cohort, have reported a higher incidence of cancer in patients with HF (mean incidence rate ratio, 1.24; 95% CI, 1.15 to 1.33; *P* < 0.001), and their prognosis was worse [36]. Thus, some authors have proposed that HF might represent an oncogenic condition [27].

## Anthracycline-induced cardiotoxicity

Anthracyclines are used extensively to treat lymphoma, sarcoma, breast cancer, and many pediatric cancers [22, 31]. About a third of women with breast cancer and half the children with cancer are treated with anthracyclines [37]. Unfortunately, anthracyclines can irreversibly damage the myocardium. The Childhood Cancer Survivor Study reported that among patients who survived at least 5 years, the cumulative incidence of cardiac disease 30 years from diagnosis was 4.8% (95% CI, 4.3 to 5.2) in 24,214 patients with a median attained age of 27.5 (range, 5.6 to 58.9) years [38]. There was a dose-response relationship between anthracycline chemotherapy and HF, with children up to age 13 years being at the greatest risk for HF after dosing similar to that of older children [38].

Anthracycline-related cardiotoxicity ranges from subclinical cardiomyopathy to HF to cardiac death. HF may occur within the first week of anthracycline treatment or even decades later [39]; however, most cases occur within the first year after treatment [19]. In fact, for at least the past two decades, anthracycline-induced HF has been a leading co-morbidity in survivors of childhood cancers [40]. In 1022 children with acute myeloid leukemia treated in the Children’s Oncology Group trial, 12% experienced cardiotoxicity (grade 2 or higher LV systolic dysfunction) during 5 years of follow-up, with more than 70% of incident events occurring during on-protocol therapy [41]. Both event-free survival (hazard ratio [HR], 1.6, 95% CI, 1.2 to 2.1; *P* = 0.004) and overall survival (HR, 1.6; 95% CI, 1.2 to 2.2; *P* = 0.005) were significantly worse in patients with documented cardiotoxicity (defined in this study as resting SF < 24% or EF < 50%).

The evidence of increased risk of CVD in cancer survivors is overwhelming. The risk is largely attributable to toxic cancer treatments, augmented by traditional cardiovascular risk factors developing later in life [32]. In a Scientific Statement from the American Heart Association (AHA) on CVD and breast cancer, the authors note that for older women, CVD poses a greater mortality threat than breast cancer itself [42]. Another AHA Scientific Statement summarizes a large amount of evidence on cardiotoxicity in children, adolescents, and young adults treated for cancer [43]. Not surprisingly, interest in preventing, minimizing, or delaying these cardiotoxic side effects remains high and continues to foster research, as well as debate.

## Primary prevention of anthracycline-induced cardiotoxicity

Strategies to prevent anthracycline-induced HF can be classified as primary or secondary [22]. Preventing cardiac damage at the time of cancer therapy (primary prevention) would be ideal, and in fact, evidence-based strategies can reduce the risk of such damage. Secondary prevention is used to refer to preventing progression to symptomatic disease, such as HF, after asymptomatic LV dysfunction has been found. The American Society of Clinical Oncology guidelines [24], endorsed by the AHA, recommend that oncologists supported by cardiologists consider several primary prevention strategies during cancer treatment.

Prevention of cardiotoxicity should be addressed by medical care providers before cancer treatment, especially with anthracyclines [44]. In addition to the specific treatment-related risks in survivors of childhood cancer, CVD risk factors are more common in these patients and present important opportunities for intervention and thus primary prevention [45]. As highlighted by the AHA Scientific Statement, Cardiovascular Risk Reduction in High-Risk Pediatric Patients, cardiovascular risk should be reduced with interventions such as counseling on maintaining appropriate weight, eating a heart-healthy diet, getting adequate exercise, and avoiding tobacco exposure [45].

### Dexrazoxane cardioprotection

Dexrazoxane is the only FDA- approved drug for preventing anthracycline-induced cardiotoxicity [46]. In August 2014, the FDA designated dexrazoxane as an orphan drug for “prevention of cardiomyopathy for children and adolescents 0 through 16 years of age treated with anthracyclines” [47]. However, citing reports that dexrazoxane might cause secondary malignant neoplasms and reduce the efficacy of doxorubicin, the European Medicines Agency (EMA) approved dexrazoxane only for women receiving doxorubicin for advanced breast cancer who require a cumulative doxorubicin dose > 300 mg/m^2^ [48]. Then, in 2017, after a careful review of dexrazoxane’s risk-benefit profile, in a rare move, the EMA overturned its earlier decision and now allows dexrazoxane to be given to children and adolescents who are likely to be treated with high cumulative doses of anthracyclines (> 300 mg/m^2^ of doxorubicin) [49, 50]**.** This recent decision allows virtually all children to receive dexrazoxane starting with the first dose of anthracycline at the discretion of the treating provider [51]. The label change announcing dexrazoxane as an approved cardiac protectant was followed with a review of the evidence by the EMA, which is posted and updated on its website [50, 51].

In a study of Wistar rats, a single dose of doxorubicin (20 mg/kg) increased circulating cardiac troponin I concentrations and decreased cardiac mass by 7.6% [52]. A 7-week regimen of doxorubicin clearly impaired the mitochondria. In a randomized, controlled trial (RCT), serum concentrations of cardiac troponin T (cTnT) (a validated biomarker of active cardiomyocyte injury) measured several times before, during, and after doxorubicin infusion in patients treated with or without dexrazoxane, found that concentrations were elevated in 20% of children who received dexrazoxane before every dose of doxorubicin but in 47% of those who did not [53]. Thus, dexrazoxane should not be withheld from any child being treated with anthracyclines, irrespective of the cumulative dose [54].

**Fig. 1:**
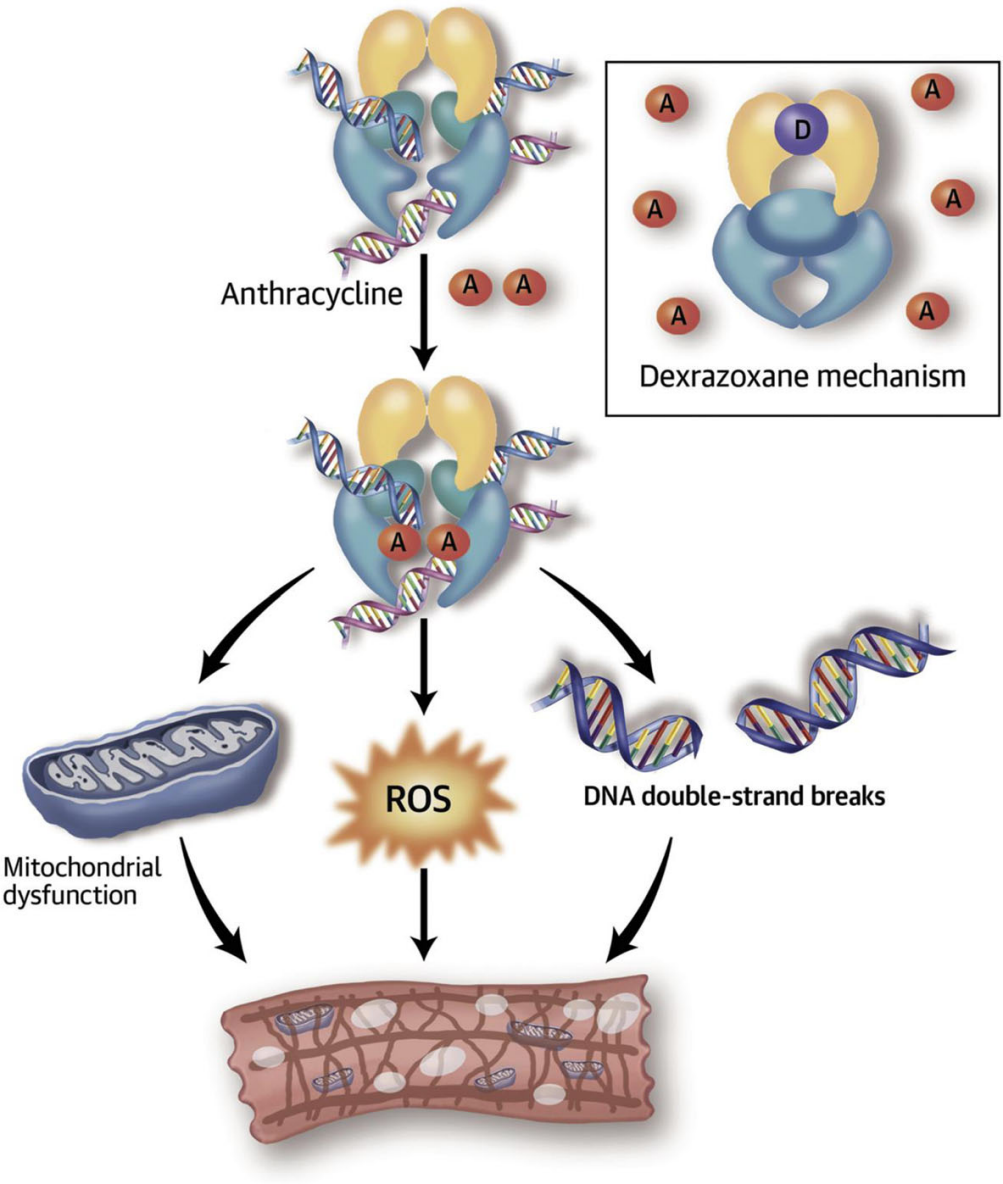
Doxorubicin (an anthracycline, A) disrupts the normal catalytic cycle of topoisomerase 2β, causing deoxyribonucleic acid (DNA) double-stranded breaks. Doxorubicin also changes the transcriptome, leading to defective mitochondrial biogenesis and increasing reactive oxygen species (ROS). As a result, cardiomyocytes show myofibrillar disarray and vacuolization. In the **inset**, dexrazoxane binds to topoisomerase 2β to prevent anthracycline binding. *Produced by permission from Vejpongsa P, Yeh ETH. J Am Coll Cardiol 2014;64:938–45*

Dexrazoxane binds iron before it enters cardiomyocytes [55, 56], which prevents the formation of the iron-anthracycline complex, thereby preventing free radical formation and thus, cardiac damage (Fig. 1). In addition, dexrazoxane can change the configuration of topoisomerase 2β, preventing anthracyclines from binding to it [22], further preventing cardiomyocyte death, mitochondrial dysfunction, and the supression of anti-oxidant gene expression [57].

Throughout its development in animal models and human studies [51, 58], dexrazoxane has consistently proven to be cardioprotective. Its acute and long-term efficacy has been confirmed in clinical studies in adults and children since the mid-1990s [59]. It has been successfully used as a cardioprotectant against anthracycline-induced cardiotoxicity in a variety of solid and hematological malignancies in adults and children receiving doxorubicin and other anthracycline drugs [49]. Multiple studies have reported that children, adolescents, and adults treated with doxorubicin and dexrazoxane have less subclinical cardiotoxicity (asymptomatic LV dysfunction) [53, 59], better LV performance [53, 60–62], and fewer cardiac events than patients not receiving dexrazoxane [63]. A Cochrane meta-analysis of 10 randomized trials on dexrazoxane (1619 pooled adults) found that dexrazoxane markedly reduced the occurrence of HF (relative risk (RR), 0.29; 95% CI, 0.20 to 0.41) [64]. Recently, five consecutive patients with preexisting, asymptomatic, LV systolic dysfunction who received anthracycline-based chemotherapy were concomitantly treated off-label with dexrazoxane, administered 30 min before each anthracycline dose, regardless of cancer type or stage [65]. In these patients, changes in LV systolic function were minimal, with a mean LVEF decreasing from 39% at baseline to 34% after chemotherapy. No patient experienced symptomatic HF or elevated cardiac troponin I or brain natriuretic peptide concentrations [65]. Meta analyses of randomized trials have suggested that dexrazoxane decreases the risk of both clinical and asymptomatic HF during and shortly after therapy [66].

As mentioned above, the authors of a prospective study of Hodgkin’s disease claimed that dexrazoxane might have increased the incidence of myelodysplastic syndrome and secondary cancers [67]. However, later and larger studies of those patients or of other patients with various cancers found no increase in the incidence of secondary malignancies in patients receiving dexrazoxane [68, 69], and dexrazoxane did not compromise long-term survival [70]. In a more recent study of 1453 patients, secondary malignancies were not related to dexrazoxane administration [71].

In a long-term study of 94 survivors of childhood cancer treated with doxorubicin (mean dose, 279 mg/m^2^), with or without dexrazoxane, those receiving only doxorubicin had a non-significant reduction in LV fractional shortening (FS; mean [SD], 33.0% [4.8%] versus 34.8% [4.6%], respectively; *P* = 0.10) but greater myocardial wall stress and dysfunction as measured by mean (SD) B-type natriuretic peptide (BNP) concentrations (18.3 [14.7] pg/mL versus 11.3 [10.6] pg/mL; *P* = 0.02) and N-terminal B-type natriuretic peptide (NT-proBNP) concentrations (64.8 [55.5] versus 44.5 [39.0] pg/mL; *P* = 0.06) [72].

In summary, the evidence for dexrazoxane’s effectiveness in reducing anthracycline-related cardiotoxicity in adults and children—without reducing antitumor efficacy and without increasing the incidence of second malignancies—is overwhelming. Research has revealed that predictive biomarkers of early anthracycline cardiotoxicity may be useful for optimizing treatment strategies through earlier implementation of cardioprotective interventions or by preventing anthracycline-associated cardiotoxicity [73]. Currently, the routine use of blood and imaging biomarkers in asymptomatic cancer patients remains limited, primarily because of a lack of evidence of utility [46].

Despite the compelling evidence of dexrazoxane’s cardioprotective efficacy, it is not routinely administered to many children, adolescents, or adults. However, the Dana-Farber Cancer Institute’s Childhood Acute Lymphoblastic Leukemia Consortium and the Children’s Oncology Group currently include dexrazoxane in their research protocols that involve anthracyclines [74]. Although dexrazoxane markedly suppresses anthracycline cardiotoxicity, its cardioprotective activity is not complete because anthracyclines have several potential cardiotoxic mechanisms, and dexrazoxane interferes with some, but not all, of these mechanisms [58, 75, 76]. In fact, there is no safe dose of anthracyclines [29]. Thus, the search for the perfect combination of primary cardioprotectants continues.

### Neurohormonal blocking drugs: Beta-blockers, angiotensin converting enzyme inhibitors, angiotensin receptor blockers, and aldosterone antagonists

#### Preclinical studies

Beta-blockers are used extensively to treat HF because of their ability to block the neurohormonal cascade that progresses to heart disease. The additional antioxidant activity of carvedilol and nebivolol has been used to justify their use for primary prevention of anthracycline cardiotoxicity. The effect of BBs for preventing specific cardiotoxic effects appears to depend on which specific receptor is being blocked. In one preclinical study in mice, the beta-1 adrenergic receptors mediated some of the acute anthracycline cardiotoxicity [77]. However, in that study, the cardioprotective rescue effect of a beta-1 receptor deletion in anthracycline-treated mice was not reproduced using the beta-1 receptor selective antagonist, metoprolol [77].

Another study evaluating metoprolol given 1 week after beginning doxorubicin therapy found no survival advantage in rats, although LV function improved [78]. On the other hand, metoprolol given to rats 3 weeks after exposure to doxorubicin temporarily down-regulated beta-adrenergic receptor density and increased attenuation of plasma norepinephrine concentrations [79]. The metoprolol-treated rats also had normal LV end-diastolic pressures. These findings suggest that metoprolol did temporarily reduce acute doxorubicin cardiotoxicity. However, the study did not address long-term cardioprotection [79]. In a third rat study, metoprolol prevented the increase in Ca^2+^-ATPase, a mechanism for doxorubicin-induced cardiotoxicity, suggesting that it has cardioprotective potential [80]. However, in contrast to the study described above [79], the density of beta-adrenergic receptors was not reduced. A 2015 study of 30 mice found that LVEF was significantly lower in those receiving doxorubicin without carvedilol than in those receiving doxorubicin with carvedilol [81].

In survivors of childhood acute lymphoblastic leukemia, doxorubicin-treated patients had an abnormally greater number of mitochondrial DNA copies per cell than did those who also received dexrazoxane [82]. This finding suggests that a higher number of mitochondrial DNA copies is required to maintain normal mitochondrial function in patients with cardiotoxicity and implicates mitochondrial damage as part of the cardiotoxicity mechanism [82]. However, in a study of rat cardiomyocytes, metoprolol did not protect against doxorubicin-induced alterations in mitochondrial DNA in regions regulating oxidative phosphorylation [83]. These contrasting results in animal studies call into question the validity of the animal models and the likelihood that metoprolol would be effective primary prevention [58].

Both ACEIs and ARBs help lower blood pressure by angiotensin blockade and thus reduce afterload, which helps cardiac function. However, two animal studies have investigated the potential of ACEIs to prevent anthracycline-induced cardiotoxicity. One randomized study in rats reported that zofenopril was more effective than enalapril or valsartan in preventing increases in doxorubicin-induced cardiomyopathy, as assessed by serum cardiac troponin I (cTnI) concentrations and histopathologic analyses [84]. This study is difficult to interpret because it did not use a true control population; rather, researchers used an ex-vivo model of stopping perfusion to an isolated rat heart after euthanasia. Thus, it is not clear whether the study evaluated a preventive effect against anthracycline damage alone, as opposed to a combined ischemic and anthracycline mechanism [84]. In another study of rats treated with doxorubicin, enalapril significantly attenuated the decrease in LVFS seen in control rats treated with only doxorubicin [85].

#### Clinical studies in adults

##### Beta-blockers

Early clinical studies have had mixed results in using BBs at the time of chemotherapy to prevent anthracycline-associated HF in adults (Table 1).

**Table 1:** Summary of studies for primary prevention of anthracycline-induced cardiotoxicity with beta-blockers, angiotensin-converting enzyme inhibitors, angiotensin receptor blockers, and aldosterone antagonists

Reference	Medications	Patients (groups), N^a^	Follow-Up, mean (SD) Months	Imaging Modality	Results by group
Avila et al. [86]	Carvedilol (3.125 mg BID increasing every 3 weeks to max 25 mg BID) vs. placebo	192 (96/96)	6	Echo	Carvedilol LVEF 65.2% → 63.9% Placebo LVEF 64.8% → 63.9% *P* = 0.84 -Lower troponin I levels in the carvedilol group (*P* = 0.003) -Lower incidence of diastolic dysfunction in the carvedilol group (*P* = 0.04)
Kalay et al. [87]	Carvedilol (12.5 mg) daily vs. placebo	50 (25/25)	5.2 (1.2)	Echo	Carvedilol: LVEF 70.5% → 69.7% Placebo: LVEF 68.9% → 52.3%† RR: 0.2 (0.03–1.59)
Tashakori et al. [88]	Carvedilol^b^ vs. control	70 (30/40)	1 week	Strain by Speckle Tracking Echo	No significant reduction in strain and strain-rate parameters after intervention, compared to control group (*P* < 0.001)
Elitok et al. [89]	Carvedilol^c^ vs. control	80 (40/40)	6	Echo	- Mean LVEF, LVFS, and LV dimensions similar before and after cancer therapy - Significantly worse LV basal septal (0.7 vs. 0.94) and lateral peak systolic strain (0.72 vs. 1.08) in control group after treatment while these measures did not differ between treatment groups at baseline. - No clinical cardiotoxic events in either group
Nabati et al. [96]	Carvedilol	91 (45/46)	6	Echo	- Carvedilol: No change in mean LVEF - Control: Mean drop of 10% LVEF Placebo group had a higher frequency of TnI concentrations > 0.05 at 30 days (48.6% vs. 24.4%, *P* = 0.03)
Jhorawat et al. [90]	Carvedilol^c^ vs. control	54 (27/27)	6	Echo	Carvedilol: LVEF 63.19% ➔ 63.88% LVFS 34% ➔ 34.6% Control: LVEF 67.27% ➔ 60.82%† LVFS 38.48% ➔ 34.6† LV end-systolic diameter Control mean (SD): 28.26 (5.50 mm➔ 31.25 (6.50) mm†) Carvedilol: unchanged
Kaya et al. [91]	Nebivolol (5 mg) daily vs. placebo ^f^	45 (27/18)	6	Echo	Nebivolol: LVEF 65.6% → 63.8% Placebo: LVEF 66.6% → 57.5%† (*P* = 0.01)
Cardinale et al. [93]	Enalapril at start of chemotherapy (prevention arm) vs. troponin triggered enalapril therapy	273 (136/137)	12	Echo	Troponin elevation incidence: Prevention group: 23% vs. Troponin triggered group 26% (*P* = 0.50) Cardiotoxicity incidence: 2 in prevention group vs. 1 in troponin-triggered group
Janbabai et al. [94]	Enalapril (17.94 [4.10] mg) vs. control	69 (34/35)	6	Echo	Δ mean LVEF from baseline at 6 months: 0.55 vs. -13.3, *P* < 0.001 In the enalapril group, tissue Doppler, E/e’ ratio, mean LVEF and cTnI and CK-MB levels were significantly unchanged compared to the controls.
Nakamae et al. [95]	Valsartan (80 mg)	40 (20/20)	7 days	Echo	Valsartan significantly inhibited the dilatation of LVDd (*P* = 0.01), elevation of BNP (*P* = 0.001), and prolongation of the QTc interval and QTc dispersion (*P* < 0.001 and *P* = 0.02, respectively)
Georgakopoulos et al. [97]	Metoprolol ^d^ vs. enalapril ^d^ vs. placebo^e^	125 (42/43/40)	31 (Longest 36)	Clinical	Cardiotoxicity incidence: Metoprolol: 1 vs. 3, not significant Enalapril: 2 vs. 3, not significant No difference in echocardiographic variables among 3 groups at 12 months Comments: -Results published as a letter not full article -Appears to be a cohort study, not a randomized trial -Cardiotoxicity not defined
Bosch et al. [11]	Enalapril (8.6 [5.9] mg) + Carvedilol (23.8 [17] mg) vs. no treatment ^f^	90 (45/45)	6	Echo and CMR	Enalapril + carvedilol: LVEF 63.3% → 62.9% Control: LVEF 64.6% → 57.9%† cTnI concentrations did not differ between 2 groups (*P* = 0.59)
Gulati et al. [12]	Candesartan (32 mg) ^g^ + metoprolol (100 mg) vs. Candesartan + placebo vs. Metoprolol ^g^ + placebo vs. Placebo + placebo	126 (30/32/32/ 32)	10–61 weeks	CMR	Δ LVEF from baseline 1. Candesartan: − 0.8 vs. -2.6%, *P* = 0.023 2. Metoprolol: − 1.6 vs. -1.8%, *P* = 0.77 Data were analyzed differently (comparing all those who received a drug to those who did not) from factorial design of the trial.
Akpek et al. [104]	Spironolactone ^h^ vs. placebo	83 (43/40)	24.0 [2.9] weeks	Echo	Spironolactone LVEF 67% → 65.7% (*P* = 0.094) Placebo LVEF 67.7% → 53.6% (*P* < 0.001) Troponin and NT-proBNP remained in normal limits. Increase in the control group was more than in the spironolactone group
Gupta et al. (PEDIATRIC) [105]	Enalapril^i^ vs. placebo	84 (44/40)	6	Echo	Enalapril LVEF 65.73% → 62.25% Placebo LVEF 64.85% → 56.15%† > 20% decrease in LVEF: Enalapril - 0 Placebo- 3 patients (8%) Higher proBNP in placebo group (*P* < 0.001) Higher cTnI level in placebo group (*P* = 0.035)
El-Shitany et al. (PEDIATRIC) [106]	Carvedilol ^j^ vs. control	50 (25/25)	After last doxorubicin dose	Echo	-FS (2D) and GPSS (2DS) significantly increased in carvedilol treated group. -Carvedilol pretreatment inhibited ADR-induced increase in plasma troponin I and LDH. (Post treatment troponin, 0.061 vs. 0.023, *P* ≤ 0.05. Post treatment, LDH 957 vs. 410, *P* ≤ 0.05)

LVEF, left ventricular ejection fraction; cTnI, cardiac troponin I; FS (2D), fractional shortening measured by 2-dimensional echocardiography; CK-MB, creatine kinase-MB; E/e’ ratio, early mitral inflow velocity: mitral annular early diastolic velocity ratio; GPSS (2DS), global peak-systolic strain measured by 2-dimensional echocardiography; ADR, doxorubicin; LDH, lactate dehydrogenase

† Statistically significant between baseline and 6 months (*P* < 0.05)

^a^ Numbers in parentheses are the numbers of patients in intervention and control or placebo groups, respectively

^b^ Carvedilol 6.25 mg daily during chemotherapy

^c^12.5 mg oral carvedilol daily for 6 months during chemotherapy

^d^Medications titrated as tolerated

^e^Medications started on the first day of chemotherapy and continued throughout the study

^f^Medications started within 1 week before the first chemotherapy cycle and continued for 6 months

^g^Starting dose was 8 mg for candesartan cilexetil and 50 mg for metoprolol succinate; target dose 32 and 100 mg, respectively

^h^ Dose was 25 mg/day started 1 week before the start of chemotherapy until 3 weeks after end of chemotherapy

^i^Dose was 0.1 mg/kg/day once a day from the first day of chemotherapy for 6 months

^j^Starting carvedilol dose was 0.1 mg kg^− 1^ d^− 1^ in two divided doses, increased weekly until reaching a dose of 1 mg/kg before the last dose of doxorubicin

The Carvedilol Effect in Preventing Chemotherapy-Induced Cardiotoxicity (CECCY trial) was an RCT of carvedilol versus placebo in 192 women with HER2-negative breast cancer. The primary endpoint of a ≥ 10% decrease in LVEF within 6 months of starting chemotherapy occurred in 14 patients (14.5%) in the carvedilol group and in 13 patients (13.5%) in the placebo group (*P* = 0.99) [86]. Changes in LVEF and BNP concentrations did not differ between groups. However, the carvedilol group had lower mean serum cTnI concentrations over 24 weeks (*P* = 0.003) and a lower incidence of LV diastolic dysfunction (*P* = 0.04). The authors concluded that carvedilol did not affect the incidence of early reductions in LVEF [86].

In a placebo-controlled clinical trial of prophylactic carvedilol used from the start of chemotherapy, after 6 months of follow-up, mean LVEF was similar to baseline in the carvedilol group (*n* = 25; 70.5 versus 69.7, respectively; *P* = 0.3) but was significantly lower in the control group (n = 25; 68.9 versus 52.3; *P* < 0.001) [87]. Doppler echocardiography showed that although the E-wave velocities in the carvedilol group were reduced, both the E-wave velocities and the E/A ratios were also significantly reduced in the control group [87]. Of the 5 patients who died, 4 were in the control group and 1 was in the carvedilol group; the difference was not significant. Unfortunately, cause of death was not discussed in the article.

In a third study, women with breast cancer treated with doxorubicin were randomly assigned to receive either a low daily dose of carvedilol (*n* = 30) or placebo (*n* = 40) before each doxorubicin dose [88]. Echocardiograms obtained 1 week after completing doxorubicin therapy revealed that LV strain and strain-rate in the women receiving carvedilol were closer to normal than were those in women receiving placebo, but mean LVEF did not differ significantly between groups [88]. No longer-term data were provided, and the differences in clinical outcomes were not reported.

A fourth randomized trial, that evaluated carvedilol in 80 women with breast cancer, had similar findings. Ventricular function, as measured by 2D-speckle tracking strain echocardiography, was better in women receiving carvedilol than in those receiving placebo after 6 months of follow-up [89]. However, the control group did not differ in any other cardiac measurements (LVEF, LVFS, or LV size) from the carvedilol group or in clinical cardiac endpoints (no patients in the trial in either group experienced a clinical cardiac event).

Another study of 54 patients tested the efficacy of carvedilol as primary prevention for doxorubicin-induced cardiomyopathy [90]. At 6 months of follow-up, LV systolic function, as measured by mean LVEF and LVFS on echocardiography, was slightly higher in the carvedilol group than in the control group (LVEF, 63.88% versus 60.82%, no *P* value was provided). Interestingly, mean LVEF and LVFS in the carvedilol group were marginally higher after treatment than before treatment. Further, the within-group differences between 6 month follow-up and baseline were not compared statisically between groups.

In a meta-analysis of carvedilol for preventing anthracycline-induced cardiotoxicity (8 RCTs, 633 pooled patients), the incidence of low LVEF was significantly lower in the carvedilol group (3.2% versus 5.8%; odds ratios [OR], 0.42; 95% CI, 0.18 to 0.99; *P* = 0.05) [91]. The authors concluded that prophylactic carvedilol in patients undergoing anthracycline treatment may reduce the incidence of LV dysfunction. However, the trials in the study had only short-term follow-ups.

Another BB, nebivolol, was also investigated in a small RCT of women with breast cancer undergoing chemotherapy in which 27 received nebivolol, 5 mg daily, and 18 received placebo [92]. After 6 months, echocardiographic measurements of LV dimensions had increased, indicating worsening, in the placebo group (*P* = 0.01) but remained unchanged in the nebivolol group (*P* = 0.93). The placebo group also had a lower mean (SD) LVEF than that of the nebivolol group (57.5% [5.6%] versus 63.8 [3.9%], respectively; *P* = 0.01) at follow-up, although the values were about equal at baseline. Serum concentrations of NT-proBNP did not change in the nebivolol group (*P* = 0.77), but they were increased in the placebo group (*P* = 0.01) [92]. The study did not report any differences in the incidence of clinical events.

##### Angiotensin inhibitors and receptor blockers

A few trials have evaluated an ARB or an ACEI for preventing anthracycline-associated cardiac dysfunction. The multicenter phase III ICOS-ONE (International CardioOncology Society-ONE) trial compared patients randomly assigned to receive enalapril at the start of chemotherapy (the prevention group) with those in whom enalapril was started only after serum troponin concentrations increased (the troponin-triggered group) [93]. The incidence of troponin elevations peaked 1 month after chemotherapy and was similar in both groups: 26% (31/136) in the prevention and 23% (36/137) in the troponin-triggered group. However, after 12 months, cardiotoxicity, defined as 10-percentage-point reduction in LVEF, with values < 50%, developed in only 3 patients, 2 in the prevention group and 1 in the troponin-triggered group. Because the outcomes did not differ, the authors recommended the troponin-triggered treatment strategy as more convenient [93].

An RCT of 69 patients receiving enalapril or placebo with anthracycline chemotherapy found no difference in mean LVEF at 6 months, although patients in the control group had significantly lower LVEF at the end of the follow-up period compared with their baseline values (LVEF: 46.31 ± 7.04 versus 59.61 ± 5.7% respectively; *P* < 0.001) [94]. This study also found that serum cTnI and creatine kinase-MB concentrations were significantly higher in the control group than in the enalapril group, suggesting some cardioprotective effect of enalapril against anthracycline-induced cardiotoxicity [94]. However, the study did not report any differences in clinical outcomes.

Another RCT evaluating only an ARB investigated the potential cardioprotective effect of valsartan in 40 patients with non-Hodgkin lymphoma treated with cyclophosphamide, doxorubicin, vincristine, and prednisone (the CHOP regimen) [95]. Valsartan significantly inhibited LV dilation (*P* = 0.01), elevations in BNP concentrations (*P* = 0.001), prolongation of the QTc interval, and QTc dispersion (*P* < 0.001 and *P* = 0.02, respectively) after chemotherapy. However, follow-up was only 1 week after initiating chemotherapy [95].

In a similar study, the same investigators randomly assigned 91 women recently diagnosed with breast cancer and treated with anthracyclines to either carvedilol or placebo and evaluated changes in LVEF 6 months after diagnosis [96]. Median reduction in LVEF from baseline was 10% in the placebo group and zero in the carvedilol group (*P* < 0.001) [96]. In addition, 30 days after therapy started, median cTnI concentration and the incidence of cTnI concentrations > 0.05 ng/mL were higher in the placebo group (48.6% versus 24.4%; *P* = 0.03) [96].

One study, published as a letter, without full methodologic details [97], compared an ACEI (metoprolol) versus BB (enalapril) versus no medication as primary prevention against anthracycline cardiotoxicity in 125 adults with lymphoma. Neither clinically important HF (which occurred in only 6 patients) nor changes in subclinical echocardiographic measures differed by group.

A few trials have investigated the efficacy of drug combinations in preventing chemotherapy-induced cardiotoxicity. In 90 patients with newly diagnosed or relapsed hematological malignancies (the OVERCOME Trial), patients treated with an ACEI (enalapril) and a BB (carvedilol) had smaller reductions in LVEF than those in untreated controls during the 6 months of observation [11]. In this study, mean LVEF, as measured by echocardiography, decreased by 3.11% in the 37 controls and by 0.17% in the intervention group (*P* = 0.04). The difference in clinical cardiac events was not reported separately, but only 2 patients experienced symptomatic HF [11]. Subsequently, a randomized, placebo-controlled, double blind trial was conducted in 120 women with breast cancer receiving post-surgery adjuvant chemotherapy with epirubicin, 5-fluorouracil, and cyclophosphamide [12]. Women were randomly assigned to one of four groups: candesartan alone, metoprolol alone, both medications, and placebo. Cardiac function was monitored with serial transthoracic echocardiograms and cMRI scans before and after cancer treatment. Scans were acquired at baseline, after the first and the final cycles of anthracycline therapy, and after trastuzumab or radiation therapy was completed. Although the candesartan group had a smaller mean decline in LVEF than did the placebo group (0.8% versus 2.6%, *P* = 0.03), metoprolol showed no evidence of being cardioprotective because the decline in LVEF was identical to that in the placebo group [12]. However, the data were analyzed by comparing all those who received any drug to those who did not, as opposed to testing the factorial design of how the medications were assigned. Thus, it was not a true test of the efficacy of combining two medications.

Among 6542 women (≥66 years old) newly diagnosed with breast cancer and identified from two population-based data sources, treatment with ACEIs or BBs was assessed as the number of prescriptions filled before or after the start of trastuzumab or anthracycline therapy. The adjusted hazard ratio for cardiotoxicity and all-cause mortality was 0.77 (95% CI, 0.62 to 0.95) in the ACEI group and 0.79 (95% CI, 0.70 to 0.90) in the BB group, compared to the hazards in the non-exposed (to either ACEI or BB) group. Starting ACEIs/BBs ≤6 months after the initiation of trastuzumab/anthracyclines and having exposed duration of ≥6 months were associated with decreased risk of cardiotoxicity and all-cause mortality [98]. Although suggesting a clinical benefit, the results are from an observational study and cannot be attributed to specific medications or drug classes or to primary, secondary, or tertiary prevention.

A meta-analysis of 14 studies (12 RCTs and 2 observational studies; 2015 pooled patients) reviewed the efficacy of several drugs for the primary prevention of chemotherapy-induced cardiotoxicity [99]. The data did not include the incidence of clinical events (e.g., hospitalizations or death from HF) and effects > 1 year [99]. The analysis did find that angiotensin antagonists (*P* < 0.001) and BBs (*P* < 0.001) prevented short-term chemotherapy-induced cardiotoxicity. However, the decreased reduction in LVEF does not necessarily mean these drugs prevented primary damage to cardiomyocytes from anthracyclines. An alternative, and more likely, explanation is that these drugs attenuated the decrease in LVEF by lowering systemic vascular resistance. Only one study in the meta-analysis, a large, single-center retrospective cohort study, considered clinical events occuring more than a year post-chemotherapy. At a median of 3.2 years after diagnosis, the 106 women with breast cancer treated with an anthracyclines, transtuzumab, or both who were taking BBs throughout treatment, had an 80% lower risk of hospitalization for HF compared to the 212 women on similar chemotherapeutic regimens who did not receive BB [100].

##### Aldosterone antagonists

Mineralocorticoid receptor antagonist blockade, such as that provide by potassium-sparing diuretics, suppresses fibrosis and improves clinical outcomes in patients with chronic HF and after myocardial infarction and supports the efficacy of aldosterone signaling in extra-renal organs [58].

In six rats, spironolactone prevented pathophysiological alterations secondary to doxorubicin-like prolongation of the QTc interval, decreased LVEF and LVFS, and increased LV end-diastolic and end-systolic dimensions (*P* < 0.05) [101]. In another study of 80 rats, although spironolactone was cardioprotective, as assessed by microscopic evidence of cardiac inflammation and fibrosis, it had no protective effect on the thoracic aorta (with respect to inflammation, fibrosis and TGF-β expression) when administered with radiotherapy and trastuzumab [102].

The effect of mineralocorticoid receptor activity and its potential as a cardioprotectant during anthracycline therapy differs in animal and human studies, perhaps because of the variety of animal models and experimental methodology used to test cardioprotective activity [58]. The effects of anthracyclines in the laboratory can, but do not always, reproduce the clinically observed cardiotoxic effects in humans [103]. Because doxorubicin cardiotoxicity develops over months or years, the applicability of results obtained by compressing the time-to-injury by administering high doses of doxorubicin to animals to understanding the chronic in vivo situation in humans is highly questionable [58].

A randomized, double-blind, placebo-controlled study of 43 women with breast cancer receiving spironolactone (25 mg/day) and 40 women on placebo treated concomitantly with doxorubicin or epirubicin suggested that spironolactone provided significant short-term cardioprotection [104]. Echocardiograms taken before chemotherapy and 3 weeks after chemotherapy showed that the decrease in LVEF was significantly less in the spironolactone group than in controls (*P* < 0.001) [104]. Similarly, LV diastolic functional grade was preserved in the spironolactone group (*P* = 0.10) but deteriorated in controls (*P* < 0.001). The study also noted that the incidence of elevated serum cardiac biomarker concentrations (creatine kinase-MB, cTnI, and NT-proBNP), total oxidative capacity, and the oxidative stress index were more pronounced in controls [104].

#### Clinical studies in children

Enalapril was evaluated as a cardioprotectant in a randomized, double blind, placebo-controlled trial of 41 children with leukemia and 43 with lymphoma who received anthracyclines (doxorubicin, daunorubicin, or both) at a cumulative dose of ≥200 mg/m^2^ [105]. The 44 children in the treatment group received enalapril, 0.1 mg/kg/day, once a day from the first day of chemotherapy for 6 months; the remaining 40 children received a placebo. After 6 months, mean (SD) LVEF had decreased in both groups, but more so in the placebo group (62 [5] versus 56 [4]; *P* < 0.001). An absolute decrease in EF ≥ 20% from baseline was seen in 3 patients in the placebo group but none in enalapril group (*P* = 0.21). Concentrations of proBNP (*P* < 0.001) and cTnI (*P* = 0.04) were higher in the placebo group [105].

In another study of 50 children with acute lymphoblastic leukemia, pretreatment of ALL children with carvedilol for 5 days before every dose of ADR caused a significant (*P* = .0015) increase (14.9%) in FS measured 1 week after the last ADR dose compared with the values after ADR treatment [106]. Carvedilol pretreatment also significantly inhibited the expected doxorubicin-induced increases in plasma cTnI and LDH concentrations see in the placebo group, suggesting a cardioprotective effect of carvedilol [106].

Carvedilol pretreatment also significantly inhibited the expected doxorubicin-induced increases in plasma cTnI and LDH concentrations see in the placebo group, suggesting a cardioprotective effect of carvedilol [106].

In summary, studies using neurohormonal blocking drugs for primary prevention of HF are not conclusively positive and have several issues. Follow-up periods are limited, sample sizes in RCTs are small, and the results are often contradictory. Further, none of the randomized trials reported a difference in the frequency of clinical events. These limitations were acknowledged in a review of needed changes in this research: 1) the duration of therapy with these drugs and 2) medications need to be evaluated for their efficacy in preventing clinical endpoints, such as HF, and not simply for their effect on surrogate endpoints, such as measures of subclinical cardiotoxicity [107]. It is important to determine whether lowering blood pressure and improving LVEF in these patients improves survival or other clinical outcomes [108, 109].

### Statins in primary prevention

One of the most widely accepted mechanisms of anthracycline cardiotoxicity is the increase in reactive oxygen species [110–113]. Statins possess “pleiotropic effects”; they decrease oxidative stress and inflammation. Anthracyclines increase oxidative stress and inflammation and thus may potentially protect against anthracycline-induced cardiac damage. In a propensity-matched cohort study, 67 women with newly diagnosed breast cancer treated with concomitant statins during anthracycline-based chemotherapy had a lower risk of HF (HR, 0.3; 95% CI, 0.1 to 0.9; *P* = 0.03) than the 134 women in the non-statin-treated comparison group [114]. Average follow-up in this study was 2.6 years after diagnosis. Another study evaluated 51 patients receiving anthracycline-based chemotherapy for lymphoma, leukemia, or breast cancer with cMRI measurements acquired before cancer therapy and 6 months after the start of therapy. After adjusting for age, sex, diabetes, hyperlipidemia, and cumulative anthracycline dose, mean (SD) pre-post differences in LVEF were small in participants receiving a statin (+ 1.1% [2.6%]), whereas the difference in those not receiving a statin declined by − 6.5% [1.5%]; *P* = 0.03) [115].

An RCT of prophylactic atorvastatin in 40 patients receiving anthracyclines found no significant difference from controls in the primary endpoint, the frequency of a LVEF < 50% after 6 months of treatment [116]. However, statin therapy resulted in a smaller decline in mean (SD) LVEF (− 1.3% [3.8%] versus − 7.9% [8.0%], *P* < 0.001) and a lesser increase in mean LV end-systolic (*P* < 0.001) and end-diastolic (*P* = 0.02) dimensions in the treatment group. An ongoing RCT (the PREVENT study, clinical trial.gov; #NCT01988571) is examining the cardioprotective effects of statin therapy in patients undergoing anthracycline-based chemotherapy. However, studies are necessary to determine whether any protective effects are truly related to statin therapy’s pleiotropic effects, secondary to decreasing ischemic cardiomyopathy, or are the concomitant effects of neurohormonal antagonist prescriptions. In any case, adults with hypercholesterolemia or at increased risk of adverse cardiovascular events (a 10-year risk for heart disease or stroke > 7.5%, as per the ACC/AHA heart risk calculator) should be treated appropriately with a statin [117].

In children**,** guidelines endorsed by the American Academy of Pediatrics recommend universal cholesterol screening in childhood [118]. Although these aggressive guidelines are well intended, their benefits and cost-effectiveness have been questioned [119]. Almost 17% of 2-to-19-year-old children were obese in 2012 [120]. Studies of obesity in cancer survivors have found similarly troubling trends, with one reporting that 13% of survivors were obese (body-mass index > 30), and that another 28% were overweight, with a body-mass index between 25 and 30 [121]. Among 893 childhood cancer survivors in The Netherlands, only girls had a significantly higher incidence of obesity [122]. Another study of more than 200 survivors from the Pediatric Long-Term Survivor Clinic at the University of Rochester found that their mean LDL-cholesterol concentration was higher than that of 70 healthy siblings [123]. In a study of direct measurements of adiposity and comparisons to contemporary controls in 170 non-Hispanic white survivors and 71 sibling controls, body fat was greater in male survivors than in controls (25.8% versus 20.7%; *P* = 0.007), as was trunk fat (26.7% versus 21.3%; *P* = 0.008) [124]. These long-term survivors of childhood cancer also have an increased incidence of dyslipidemia, hypercholesterolemia, and hypertriglyceridemia [125–127].

Another study compared 156 survivors, either exposed or unexposed to anthracyclines and cardiac radiation, to 76 healthy sibling controls. Mean fasting serum concentrations of non-high-density lipoprotein cholesterol were higher in exposed survivors than in unexposed survivors and controls (126.5 and 121.1 mg/dL, respectively, versus 109.8 mg/dL), as were insulin concentrations (10.4 and 10.5 μU/mL, respectively, versus 8.2 μU/mL) [123]. Several epidemiological studies have reported an increased incidence of the metabolic syndrome and CVD in childhood cancer survivors [128].

These increased risk factors clearly predispose this group to future health problems. Although theoretically, statins may be cardioprotective in patients with several cardiovascular risk factors, the benefits and risks remain unclear in the absence of any long-term studies in these patients.

### Exercise in primary prevention

Survivors of childhood cancers are more likely to be physically inactive than their siblings and less likely to meet recommended physical activity guidelines [129, 130]. In a study of 72 survivors of childhood cancers with a mix of diagnoses but of similar age and receiving similar chemotherapeutic drugs, at a mean of 13.4 years after their cancer diagnosis (range, 4.5 to 31.6 years), survivors had significantly lower exercise capacity (VO_2_ max), less endurance (time to peak exercise), and lower anaerobic thresholds than did their 32 siblings [131]. Peak oxygen uptake (VO_2_ max) during exercise testing was significantly reduced in 30% of survivors, [132] and the long-term exercise capacity and fitness level in these survivors were poor [131, 133]. Cancer survivors often rank fatigue as their primary concern. A weight-loss trial in breast cancer survivors found that improvements in vitality were primarily associated with increases in physical activity rather than changes in body mass index [134]. Regular physical activity may also improve nutritional and cardiac conditions.

In a single-center, prospective study of 100 childhood cancer survivors with normal baseline LVEFs, after 10 years of follow-up, the LV and right ventricular systolic and diastolic myocardial responses to exercise were similar to those of 51 healthy controls [135].

Exercise training for breast cancer survivors is safe and has several physiological and psychological benefits [136]. Some groups have recommended that cancer survivors engage in moderate aerobic exercise 150 min/week or vigorous aerobic exercise 75 min/week [136, 137]. A recent meta-analysis of the benefit of exercise in cancer patients and survivors further refined this recommendation and concluded that although exercise should be encouraged for most cancer patients, targeting specific subgroups may be more beneficial and cost effective [138]. In breast cancer survivors, exercise prescriptions based on heart rate reserve are too intense, and those based on VO_2_ max are slightly less intense than optimal. The study found that the recommended percentages for maximal heart rate appear valid [137].

Survivors of childhood cancer should be encouraged to exercise regularly to improve exercise capacity, weight, mental status, and cardiometabolic risk [131]. However, survivors with certain characteristics, such as restrictive cardiomyopathy, should be closely monitored because unsupervised exercise puts them at risk for pulmonary congestion and arrhythmias [131, 139].

Whether physical conditioning and rehabilitation programs are potentially detrimental to some survivors or help only a subset needs to be determined. For safety, exercise prescriptions should be based on the condition of the individual patient, rather than for a group of survivors, and should be periodically reevaluated because survivors’ health changes over time [131, 139]. Hopefully, appropriate and safe increases in physical activity will decrease survivors’ cardiovascular risk [129, 140]. A recent Scientific Statement from the American Heart Association promotes the use of cardiac rehabilitation to provide structured exercise to cancer patients and survivors [141]. This AHA Scientific Statement also discusses the need for research to fully develop and implement a multimodal model of the cardio-oncology rehabilitation.

## Secondary prevention of anthracycline-induced cardiotoxicity

Managing asymptomatic anthracycline cardiotoxicity and preventing symptoms, as well as florid HF and death, is the definition of secondary prevention. There are no evidence-based guidelines for monitoring cardiotoxicity during and after anticancer therapies in adults or children. Although expert consensus guidelines have been published, the efficacy of specific regimens has not been determined, and recommendations from different groups are not consistent [20, 142]. This lack of evidence, even for screening, as well as inconsistent recommendations, are the first challenges for clinicians in attempting secondary prevention.

### Neurohormonal blocking drugs and implantable devices

#### Preclinical studies

In a rabbit model of doxorubicin cardiotoxicity, metoprolol did not change or reduce the frequency or severity of arrhythmias, in contrast to treatment with carvedilol, which reduced the arrhythmic risk of anthracycline cardiotoxicity [143].

#### Clinical studies in adults

The most accepted current efforts in cardioprotection relate to recognizing early impaired LV function, which allows early interventions, such as administering a drug to attenuate or reverse the effect. The European Society for Medical Oncology clinical practice guidelines recommend that all adult cancer patients with HF and an LVEF < 40% be treated with ACEIs in combination with a BB, unless specifically contraindicated. According to Strength-of-Recommendation Taxonomy [SORT] criteria, this recommendation is based on the best-quality evidence: level 1, grade A) [20]. However, these recommendations are based largely on cardiology practice in the general population and on only a few small studies examining the utility of these drugs in cancer patients and survivors.

A retrospective study of 10 adults with anthracycline-induced cardiomyopathy (an LVEF of ≤45%) found an early benefit of the BB, metoprolol, when compared to 16 age-and-sex-matched controls with idiopathic dilated cardiomyopathy also receiving BBs [144]. The mean pre-treatment LVEF of 28% improved to 41% (*P* = 0.04) in the 10 patients at an average of 8 months after beginning treatment with metoprolol [144].

In an RCT, 114 cancer patients with elevated serum cTnI concentrations were randomly assigned to either enalapril 20 mg/day or to no treatment for 1 month after high-dose chemotherapy [145]. After 12 months, cardiotoxicity (defined as an absolute decrease to < 50% or a decrease of ≥10% in resting LVEF from baseline) was detected in 25 of 58 patients not receiving enalapril but in none of 56 patients treated with enalapril [145]. In addition, the cumulative number of adverse cardiac events (sudden death, death from a cardiac cause, acute pulmonary edema, overt HF, and life-threatening arrhythmias requiring treatment) in patients treated with enalapril was much lower than that in controls, with HF itself occurring in 0 and 24% (*P* < 0.001) in each group respectively [145].

The International Cardio Oncology Society-ONE study, mentioned above, compared the effects of enalapril for primary prevention of cardiotoxicity to its effect in secondary prevention (when administered after a rise in troponin concentrations) [93]. The two enalapril treatment strategies did not differ in their ability to prevent myocardial injury, as detected by increases in troponin concentrations, leading the authors to advocate enalapril for secondary prevention [93].

Whether BBs or ACEIs truly attenuate anthracycline-associated cardiotoxicity has not been established. Although some short-term responses are statistically significant, these could be due to the hemodynamic effects of afterload reduction and true “prevention” of cardiotoxicity may not have occurred. Longer follow-ups are necessary, as is identifying the primary mechanism of cardiotoxicity. The causes of the endpoints are confounded by other factors, making it impossible for any of these studies to truly represent “primary prevention.”

A prospective study of 201 consecutive patients (mean [SD] age, 53 [12] years; 149 women) with anthracycline-induced cardiomyopathy (LVEF ≤45%), with or without HF (74% of patients in NYHA functional class I or II), found that early administration of enalapril, and when possible carvedilol, was associated with better LVEF recovery and fewer cardiac events during the mean (SD) follow-up period of 36 (27) months; range, 12 to 96 months [146]. Responders to cardioprotective therapy, defined a complete recovery of LVEF, had lower rates of cumulative cardiac events (sudden death, cardiac death, acute pulmonary edema, HF requiring hospitalization, life-threatening arrhythmias, and conduction disorders requiring pacemaker implantation) than did partial (some recovery of LVEF) and non-responders (no recovery of LVEF): 5, 31, and 29%, respectively (*P* < 0.001) [146].

In the only known RCT to date, prophylactic implantation of cardioverter-defibrillators for primary prevention of sudden cardiac death has not benefited patients with non-ischemic cardiomyopathy (LVEF ≤35%) and symptomatic systolic HF, in contrast to the successes in patients with ischemic-cardiomyopathy [147]. This result challenges the utility of these devices in patients with chemotherapy-induced cardiomyopathy. It should be noted that 58% of patients in this trial received cardiac resynchronization therapy. In contrast, a small, single-center study of 18 consecutive patients with anthracycline-induced cardiomyopathy reported improvements in echocardiographic measurements and clinical benefit with cardiac resynchronization therapy not seen in patients with non-ischemic cardiomyopathy [148]. However, the data is limited. An ongoing multicenter, non-randomized, prospective observational study, the Multicenter Automatic Defibrillator Implantation Trial - Chemotherapy-Induced Cardiomyopathy (MADIT-CHIC; NCT02164721), is testing the effectiveness of resynchronization therapy in this population with a target enrollment of 100 patients.

#### Clinical studies in children

Only two RCTs have evaluated the use of medications to prevent asymptomatic cardiotoxicity in childhood cancer survivors from progressing to clinically evident cardiotoxicity, though multiple case series have been reported. In three children with congestive HF after receiving doxorubicin, metoprolol improved symptoms and echocardiographic measurements of LV structure and function over 5 to 30 months [149]. In another case series, 22 childhood cancer survivors (median age, 14.8; range 6.4 to 21.6 years), echocardiograms acquired before and during therapy with ACEs or ARBs were analyzed retrospectively with two-dimensional speckle tracking [150]. Mean global longitudinal strain (*P* = 0.002), global circumferential strain (*P* = 0.03), longitudinal strain rate (*P* = 0.02), and circumferential strain rate (*P* = 0.03) improved on therapy. Improvement was maintained for > 1 year on ACEI or ARB (*P* = 0.02) [150]. However, the indications for administering ACEIs or ARBs were not given, so the number of patients in whom cardiac symptoms actually prompted therapy is unknown.

In a review of 18 childhood cancer survivors with symptomatic and asymptomatic anthracycline-associated cardiomyopathy, enalapril temporarily improved LV structure and function [151]. However, enalapril did not prevent disease progression; it merely delayed it for 6 to 10 years for patients with asymptomatic LV dysfunction at the start of treatment before returning to baseline [151]. For enalapril-treated patients with HF on this study, the benefit was only for 2 to 6 years. All 6 patients with HF progressed to cardiac transplantation or cardiac death within 2 to 6 years. Enalapril also did not prevent progressive LV wall thinning, the primary defect that increases LV afterload and decreases LVFS in survivors of childhood cancer [151]. In other words, enalapril did not address the primary defect of an inappropriately thin LV wall, it just reduced LV afterload secondary to a short-term lowering of diastolic blood pressure and LV dilation [151].

Many clinicians have justified using ACEIs for secondary prevention in childhood cancer survivors with the results of the ACEI After Anthracycline (AAA) trial. This RCT of enalapril in 146 childhood cancer survivors had only one significant finding: a reduction in LV end-systolic stress in the first year of therapy, which was entirely attributable to a reduction in blood pressure attributed to ACEIs (*P* = 0.04) [152]. However, enalapril was associated with a significantly higher risk of dizziness or hypotension (RR, 7.17; 95% CI, 1.71 to 30.17) and fatigue (*P* = 0.01) [152].

A Cochrane review found two randomized trials evaluating medical interventions for anthracycline-induced cardiotoxicity in childhood cancer survivors [153]. One was the ACEI After Anthracycline (AAA) study mentioned above, and the other was a study comparing 2 weeks of treatment with phosphocreatine against controls not receiving phosphocreatine. The authors concluded that, although enalapril may temporarily improve LV function, whether it improves long-term clinical outcomes was unclear [153].

The Children’s Oncology Group is currently studying the effects of a 2-year course of carvedilol in an RCT of 250 childhood cancer survivors diagnosed before age 21 years and previously treated with high cumulative doses of anthracyclines (≥300 mg/m^2^) [154]. The primary objective is to determine the effect of carvedilol on echocardiographic measures of cardiac remodeling and the risk of HF, including the LV wall thickness:dimension ratio, LVEF, LV volume, and serum biomarker concentrations (natriuretic peptides, galectin-3) that are associated with increased risk for HF.

The studies reviewed above, with medications such as BBs, ACEIs, and ARBs given for primary or secondary prevention, may show occasional short-term improvements in LVEF. However, no studies have found long-term differences in clinical outcomes. In contrast, evidence-supported cardioprotective therapies, such as dexrazoxane, started before and maintained during chemotherapy, allow life-saving chemotherapies to be administered while limiting adverse cardiovascular events.

### Non-anthracycline anti-neoplastic drugs (targeted therapy and immunotherapy)

In the past two decades, a better understanding of the molecular pathways involved in tumor progression has led to more selective, mechanism-based therapies [155]. An explosion of new cancer therapies has revolutionized therapy and markedly improved cancer prognosis. However, some of these therapies have an assortment of cardiovascular complications [156]. In addition, strategies for preventing these cardiotoxicities are not as well studied as are those for conventional therapies, such as anthracycline-induced cardiomyopathy.

## Problems with the long-term use of BETA-blockers

Although historically, the long-term use of BBs was thought to decrease the risk of certain types of cancers by weakening norepinephrine signaling, the opposite may occur [157]. Additionally, BBs have displayed pro-angiogenic activity through a mechanism independent of their ability to antagonize catecholamine action. β-adrenergic receptor signaling facilitates VEGF-mediated angiogenesis [158]. This newly recognized signaling pathway is concerning because it may affect the prognosis of patients with solid cancers, in whom this signaling may facilitate tumor angiogenesis.

The mechanistic concerns about BBs have been apparent in at least three studies. At a median follow-up of 6.6 years, 8100 survivors of colorectal cancer showed no major improvements in survival after taking BBs [159]. In fact, a cumulative duration of treatment from 1 to 12 months was significantly associated with increased overall mortality (RR, 1.20; 95% CI, 1.03 to 1.39) [159]. In a nationwide Danish cohort of 18,733 women treated for breast cancer, metoprolol was associated with increased recurrence rates (adjusted HR, 1.5; 95% CI, 1.2 to 1.8) [157]. Finally, a population-based, case-control study revealed that the long-term (more than 6 years) use of BBs was associated with a significantly higher risk of stage IV colorectal cancer (OR, 2.02; 95% CI, 1.25 to 3.27) [160]. However, BB use was not associated with the overall incidence of colorectal cancer, the primary outcome of the study [160]. Taken together, these studies suggest that long-term BB use in survivors has no adverse effects on cancer relapse or secondary cancers. However, these studies were not performed in survivors of childhood cancers, who are known to have an increased susceptibility to an oncologic relapse or the development of a secondary malignancy.

## Problems with long-term use of angiotensin receptor blockers and angiotensin converting enzyme inhibitors

Studies have raised concerns about the long-term use of ARBs and ACEIs in cancer survivors. The Candesartan in Heart Failure Assessment of Reduction in Mortality and Morbidity (CHARM) study, that compared candesartan with placebo in patients with chronic HF, reported a 42% higher incidence of fatal neoplastic diseases during candesartan treatment [161]. In a meta-analysis of five trials with cancer recurrence as a pre-specified endpoint, the 61,590 pooled patients randomly assigned to receive ARBs had a significantly higher risk of new cancer occurrence than that of controls (RR, 1.11; 95% CI, 1.04 to 1.18, *P* = 0.001) [162]. This meta-anaylsis concluded that ARBs are associated with a modestly increased (9%) risk of new cancer diagnoses [162]. A second meta-analysis, performed 6 years later and including 19 studies (148,333 patients), also reported an 8% higher risk of cancer with ARBs than with placebo, but not if the control group received an ACEI [163]. This result provides indirect evidence that ACEIs and ARBs both increase the risk of cancer when compared to placebo. Recently, the US Food and Drug Administration recalled the ARB losartan potassium hydrochlorothiazide over concerns that an impurity might be carcinogenic [164].

A cohort study found a higher incidence of basal and squamous cell carcinoma in patients with a documented order for an ACEI, ARB, or thiazide and no history of skin cancer [165]. The control group consisted of matched individuals from the same medical practice without documented exposure to these drugs. This potential higher cancer rate may be more of a concern to cancer survivors, who are already at increased risk for relapsed primary or secondary malignancies [166]. Indeed, the national Danish cohort of women with breast cancer revealed a small, but not statistically significant, increase in cancer recurrence with ACEI use (HR, 1.2; 95% CI, 0.97 to 1.4). On the other hand, a meta-analysis of 14 trials with cancer data on 61,774 pooled patients found that ACEIs did not significantly affect the occurrence of cancer or cancer-related deaths in the general population [167].

In a population-based study of 992,061 patients newly treated with antihypertensive drugs over 20 years [167], ACEIs were associated with a higher risk of lung cancer than that in patients receiving ARBs (incidence rate, 1.6 versus 1.2 per 1000 person years, respectively; HR 1.14, 95% CI; 1.01 to 1.29). Hazard ratios gradually increased with longer durations of use, with a statistically significant association evident after 5 years of use (HR, 1.22; 95% CI, 1.06 to 1.40) and peaking after more than 10 years of use (HR, 1.31; 95% CI, 1.08 to 1.59) [168].

## Problems with the long-term use of aldosterone antagonists

Aldosterone antagonists are associated with acute renal failure and hyperkalemia, both of which can be particularly challenging in patients undergoing cancer therapy, given their increased risk of kidney dysfunction and electrolyte imbalance in the setting of malignancy and chemotherapy. Non-selective drugs, such as spironolactone, have estrogenic effects that increase the risk of painful gynecomastia in up to 50% of patients [169]. Although spironolactone has been associated with a reduced incidence of prostate cancer [170], an increased risk of breast cancer with chronic use is a concern. However, convincing data from a retrospective cohort study of 1.3 million women followed for 4 years suggests that long-term management of cardiovascular conditions with spironolactone does not increase the risk of breast cancer [171].

## Discussion

Neurohormonal antagonists are routinely used in adults with HF and are now being increasingly used—without strong evidence—in the fragile population of childhood and adult cancer survivors as a response to asymptomatic LV dysfunction without clinical HF (secondary prevention) as well as for primary prevention of cardiotoxicity [31, 58]. The literature specific to using these medications to prevent anthracycline-associated LV dysfunction in both adults and children is limited. Most studies have small samples and short follow-ups, with some of the longest being 12 months after the end of therapy. Few studies, as mentioned above, have reported clinical events as a primary outcome and instead have used subclinical measures, albeit markers validated in the general population. Thus, the “success” of the primary prevention studies should be questioned [58, 93]. Another review concluded that studies with longer follow-ups tended to show that treatment with β-blockers and ACEI or ARBs do not prevent chemotherapy-induced cardiotoxicity [13].

Errors in equating LV dysfunction with myocardial damage may also be a factor in these studies. Alterations in LV function, identified as LV dysfunction, can often be secondary to a change in the loading conditions of the heart. Thus, changes in loading conditions do not always equate to myocardial damage and the “true effect” of the medications may be confounded. Results are also complicated by the fact that these studies tested several different dose schedules and age groups. Additionally, some trials and meta-analyses lacked rigorously defined clinical endpoints or used endpoints that combined subclinical findings and clinical events [58]. These studies show only a temporary improvement in LV echocardiographic variables and a lack of increase in cardiac biomarker concentrations, but no differences in the incidence of clinical events.

In contrast, some evidence indicates clinically beneficial effects of neurohormonal antagonist drugs, particularly ACEIs and BBs, administered for secondary prevention in patients with anthracycline-induced cardiomyopathy. In most of these studies, treatment with one or more neurohormonal antagonist was started in response to an asymptomatic elevation of cTnI concentrations or reduction in LVEF. Some studies suggest that the early introduction of cardioprotective therapy is associated with higher chances of LVEF recovery and fewer clinical cardiac events including, HF, arrhythmias, and death [146]. Although neurohormonal therapy is effective for secondary prevention after asymptomatic or subclinical cardiotoxicity develops, many non-specific symptoms are attributed to cancer and its treatment, even though they are often indistinguishable from those of clinical HF, meaning that many patients may be misclassified. No study of ACEIs, ARBs, aldosterone antagonists, or BBs in survivors of adult cancers has reported persuasive evidence that these drugs improve survival or quality-of-life when used for either primary or secondary prevention.

Even fewer studies have addressed the use of BBs or ACEIs in children. Any adverse effects of long-term therapy place survivors of childhood cancers at higher risk simply because of their markedly longer anticipated survival [109]. Patients using these medications long-term may also be at increased risk for other potential adverse events from these medications.

Although this lack of evidence may be explained by underpowered studies, the lack of benefit from standard heart failure medications may also be the result of fundamental differences between cardiomyopathy caused by chemotherapy-induced cardiotoxicity and that with other causes, such as ischemic, post-infectious, and idiopathic dilated cardiomyopathy [172]. The 10-year survival rate for pediatric cancers is now more than 80% [3]. Although these children are a small percentage of survivors, they have the longest potential life spans. Thus, preventing cardiotoxicity may be most important to them as individuals and as a population, especially considering the societal cost of medical care.

In long-term survivors of childhood cancers who have been treated with anthracyclines, the dominant clinical pattern is a progression from a dilated cardiomyopathy to a restrictive cardiomyopathy with increasing follow-up, whereas in contrast, adults treated with anthracyclines develop a chronic dilated, hypokinetic cardiomyopathy [173]. Restrictive cardiomyopathy is typically less responsive to the neurohormonal drugs used to treat other cardiomyopathies [109]. In most patients with dilated cardiomyopathy, ACEIs induce reverse-ventricular remodeling, reducing LV volume and improving the LV mass-to-volume ratio, further reducing LV wall stress and improving LV function. In childhood cancer survivors with anthracycline-induced cardiomyopathy treated with ACEIs, ventricular remodeling in response to the fall in LV wall stress associated with afterload reducing agents is notably absent. In the two studies of children mentioned above [151, 152], despite a marked reduction in LV wall stress, LV size and thickness did not change. This lack of improvement is most likely caused by the minimal, non-progressive LV dilation in these patients, which is more characteristic of a restrictive cardiomyopathy, a disease class that does not benefit from ACEI therapy [172]. Further, many participants in the ACEI After Anthracycline (AAA) trial also received cardiac radiation, which is also associated with restrictive cardiomyopathy [152]. Admittedly, to what extent this restrictive predominance applies to survivors treated as adults is unclear. Yet, this predominance of restrictive cardiomyopathy does raise the concern that in adults, anthracycline-related cardiomyopathy is not the same as ischemic cardiomyopathy, as well as the concern that the neurohormonal blocking agents will not be effective [109].

Current data do not address whether the potential adverse effects of neurohormonal antagonists in patients with anthracycline-induced cardiomyopathy exceeds that in patients with other forms of dilated cardiomyopathy [109]. Thus, the wisdom of recommending medications based on the applicability of trials in other patient groups has been and should continue to be questioned [109]. Survivors of childhood cancers are at increased global risk for premature atherosclerotic heart disease, a risk that may also be affected by ACEI therapy [174]. Angiotensin converting enzyme inhibitors may cause fetal kidney abnormalities if taken during pregnancy, which may be a particular concern for female childhood cancer survivors [175].

The unknown effects of chronic neurohormonal suppression or other side effects of long-term BB therapy in this population are also a concern. Dizziness, hypotension, and fatigue were common problems for enalapril-treated participants in the ACEI After Anthracycline (AAA) trial and may be aggravated in this fragile population [152]. The considerable cost of an unproven, potentially life-long therapy, especially in young patients with large medical expenses and possible lifetime insurance limits, makes this therapy difficult to justify [109]. Potentially life-long medication use for asymptomatic patients also raises daily adherence issues. The potential for healthy, asymptomatic survivors to feel or be treated differently from their peers for taking chronic medications may increase the likelihood of these children feeling like “cardiac cripples” [109].

Therefore, we believe the evidence argues against routinely using these medications in patients with potential chemotherapy-induced cardiomyopathy, particularly for primary cardioprotection. Once subclinical or asymptomatic cardiotoxicity appear, as indicated by elevations in cardiac biomarker concentrations or as increases in global longitudinal strain or LVEF reduction without clinical HF, the decision to use neurohormonal antagonists for secondary prevention needs to be individualized after careful consideration of the patient’s cardiac risk profile, type of anti-neoplastic therapy received, and personal preferences.

The above cautions notwithstanding, as in primary prevention, dexrazoxane may still be useful, even in secondary prevention. However, dexrazoxane should be compared to BBs, ACEIs, aldosterone antagonists, and ARBs in RCTs because quality-of-life remains paramount in long-term survivors, especially those with pre-existing risk factors.

Strategies to minimize cardiotoxicity during treatment are crucial to preventing lasting effects on health and quality-of-life. Because the long-term use of neurohormonal antagonists for primary or secondary prevention of cardiotoxicity in cancer patients and survivors has potential risks with unproven benefits, larger and longer clinical trials are needed to verify the efficacy and tolerability of these drugs. The short-term results in some of the studies reviewed could be used to justify further exploration of these drugs for cardioprotection in patients with cancer. However, longer, adequately powered, well-designed clinical trials with clinical endpoints, including comparative analysis with existing effective medications, such as dexrazoxane, are required [176].

Arguably, one of the biggest challenges in the field of cardio-oncology is selecting appropriate endpoints. A single cardiology endpoint for a patient without symptomatic CVD is not likely to be as clinically meaningful as are oncologic, symptomatic CVD, or quality-of-life endpoints in this population. In children, studies using clinical endpoints, such as death, will likely be unable to detect efficacy because the time-to-event for these endpoints is decades long and thus are not feasible for prospective studies. Validated cardiac biomarkers should be incorporated into studies as surrogate endpoints when assessing reductions of anthracycline cardiotoxicity [58]. A combined “imaging-and-biomarker” approach has been suggested to increase the predictive value over that of a single indicator [18]. Finally, with all the side effects and potential risks of life-long use of these medications, recognizing that quality-of-life is an important end-point for these trials is important.

As the number of cancer survivors grow, particularly older patients with comorbid CVD receiving treatment for cancer, oncologists are increasingly reliant on CV specialists to risk-stratify and to address a myriad of comorbidities and the adverse effects of cancer therapeutics. Although the field of cardio-oncology is emerging at a fast pace, it is mainly restricted to a limited number of academic centers. To usher patients safely through cancer care, cardio-oncology care needs to expand simultaneously and hence training the next-generation of physicians in cardio-oncology is necessary [177].

## Conclusion

Cardio-oncology is a broad, active, and new field of medicine. Here, we have summarized the strengths, weaknesses, opportunities, and threats in the field as identified in the most important published studies with special emphasis on the prevention of anthracycline-induced cardiotoxicity.

Cancer and the resultant cardiotoxicity from both conventional and contemporary therapy substantially affect an increasing number of survivors. The optimal strategy for preventing and managing chemotherapy-induced cardiotoxicity remains unknown. We would contend that the routine use of neurohormonal antagonists for primary cardioprotection in this population is not currently justified, given only marginal benefits and associated adverse events, particularly with long-term use. Their use for secondary prevention in patients with subclinical cardiotoxicity should be individualized and carefully considered. On the other hand, dexrazoxane provides effective primary cardioprotection against anthracycline-induced cardiotoxicity, and its use beyond the current FDA-approved indications should be investigated further. Longitudinal studies are needed to determine the prognostic value of subclinical markers of treatment-related cardiovascular injury on the long-term risk of CVD.

## Data Availability

Not applicable.

## Abbreviations

ACEI: Angiotensin converting enzyme inhibitor
AHA: American Heart Association
ARB: Angiotensin receptor blocker
BB: Beta-blocker
BNP: B-type natriuretic peptide
cMRI: Cardiac magnetic resonance imaging
cTNI: Cardiac Troponin I
cTnT: Cardiac Troponin T
CVD: Cardiovascular disease
DCM: Dilated cardiomyopathy
EMA: European Medicines Agency
FDA: Food and Drug Administration
FS: Fractional shortening
HF: Heart failure
LV: Left ventricle
LVEF: Left ventricular ejection fraction
LVFS: Left ventricular fractional shortening
NT-proBNP: N-terminal B-type natriuretic peptide
RCT: Randomized controlled trial
